# *catena*-Poly[[[tetra­kis­(cyanido-κ*C*)tungstate(IV)]-di-μ-cyanido-κ^4^ *C*:*N*-bis­[diaqua­(2,2′-bipyridyl-κ^2^ *N*,*N*′)manganese(II)]-di-μ-cyanido-κ^4^ *N*:*C*] hexa­hydrate]

**DOI:** 10.1107/S1600536811012451

**Published:** 2011-05-07

**Authors:** Noriaki Ozaki, Ryo Yamada, Koji Nakabayashi, Shin-ichi Ohkoshi

**Affiliations:** a Department of Chemistry, School of Science, University of Tokyo, 7-3-1 Hongo, Bunkyo-Ku, Tokyo 113-0033, Japan

## Abstract

The polymeric title compound, {[Mn^II^_2_W^IV^(CN)_8_(C_10_H_8_N_2_)_2_(H_2_O)_4_]·6H_2_O}_*n*_, has a one-dimensional cyanide-bridged Mn^II^–W^IV^ bimetallic assembly. The coordination geometry of the W^IV^ atom is eight-coordinate square-anti­prismatic and that of each of the Mn^II^ atoms is six-coordinate distorted octa­hedral. Two pairs of CN ligands of W(CN)_8_ are bridged to two Mn^II^ atoms, the remaining CN ligands being terminal. Each Mn^II^ atom is additionally coordinated by a bidentate 2,2′-bipyridyl ligand and two water mol­ecules. The crystal structure is stabilized by O—H⋯O and O—H⋯N hydrogen bonds.

## Related literature

For general background to octa­cyanido­tungstates as magnetic materials, see: Ohkoshi *et al.* (2007 ▶, 2008 ▶); Sieklucka *et al.* (2009 ▶). For related octa­cyanido­tungstate structures, see: Herrera *et al.* (2003 ▶); Leipoldt *et al.* (1994 ▶); Sieklucka *et al.* (2000 ▶).
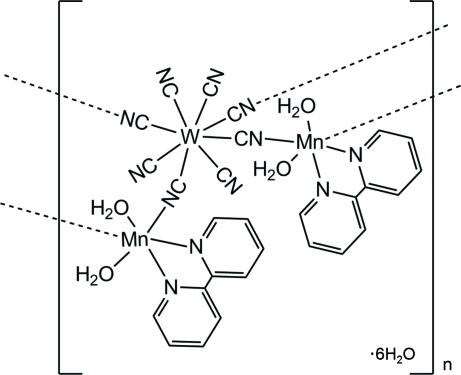


## Experimental

### 

#### Crystal data

[Mn_2_W(CN)_8_(C_10_H_8_N_2_)_2_(H_2_O)_4_]·6H_2_O
*M* *_r_* = 992.40
Monoclinic, 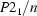

*a* = 11.6673 (3) Å
*b* = 15.5511 (4) Å
*c* = 21.4339 (7) Å
β = 99.213 (1)°
*V* = 3838.78 (18) Å^3^
*Z* = 4
Mo *K*α radiation
μ = 3.70 mm^−1^
*T* = 90 K
0.90 × 0.20 × 0.02 mm

#### Data collection

Rigaku R-AXIS RAPID diffractometer
Absorption correction: multi-scan (*ABSCOR*; Higashi, 1995 ▶) *T*_min_ = 0.135, *T*_max_ = 0.930
33108 measured reflections
8606 independent reflections
7342 reflections with *I* > 2σ(*I*)
*R*_int_ = 0.062

#### Refinement

*R*[*F* ^2^ > 2σ(*F* ^2^)] = 0.036
*wR*(*F* ^2^) = 0.079
*S* = 1.03
8606 reflections
533 parameters
18 restraints
H atoms treated by a mixture of independent and constrained refinement
Δρ_max_ = 2.47 e Å^−3^
Δρ_min_ = −1.92 e Å^−3^

### 

Data collection: *PROCESS-AUTO* (Rigaku, 2007 ▶); cell refinement: *PROCESS-AUTO*; data reduction: *CrystalStructure* (Molecular Structure Corporation & Rigaku, 2007 ▶); program(s) used to solve structure: *SHELXS97* (Sheldrick, 2008 ▶); program(s) used to refine structure: *SHELXL97* (Sheldrick, 2008 ▶); molecular graphics: *ORTEP-3* (Farrugia, 1997 ▶) and *VESTA* (Momma & Izumi, 2006 ▶); software used to prepare material for publication: *CrystalStructure*.

## Supplementary Material

Crystal structure: contains datablocks I, global. DOI: 10.1107/S1600536811012451/tk2734sup1.cif

Structure factors: contains datablocks I. DOI: 10.1107/S1600536811012451/tk2734Isup2.hkl

Additional supplementary materials:  crystallographic information; 3D view; checkCIF report

## Figures and Tables

**Table 1:** Hydrogen-bond geometry (Å, °)

*D*—H⋯*A*	*D*—H	H⋯*A*	*D*⋯*A*	*D*—H⋯*A*
O1—H1⋯O10^i^	0.84 (2)	2.02 (2)	2.822 (5)	162 (5)
O1—H2⋯O9^ii^	0.84 (2)	2.04 (3)	2.834 (4)	156 (5)
O2—H3⋯O6^iii^	0.83 (2)	1.87 (2)	2.703 (4)	173 (5)
O2—H4⋯N6^i^	0.82 (2)	2.25 (2)	3.052 (4)	165 (5)
O3—H6⋯O6^iv^	0.83 (2)	1.92 (2)	2.744 (4)	176 (5)
O3—H5⋯N5^iv^	0.82 (2)	2.48 (3)	3.238 (4)	153 (4)
O4—H8⋯O5^iv^	0.83 (2)	2.12 (2)	2.907 (5)	159 (4)
O4—H7⋯O9^v^	0.84 (2)	1.89 (2)	2.722 (4)	174 (4)
O5—H30⋯N6^vi^	0.84 (2)	2.13 (2)	2.946 (5)	164 (5)
O5—H29⋯O1^i^	0.84 (2)	2.32 (3)	3.111 (4)	157 (5)
O6—H32⋯O7	0.83 (2)	1.97 (2)	2.773 (4)	162 (5)
O6—H31⋯O5	0.83 (2)	1.97 (3)	2.753 (4)	157 (4)
O7—H34⋯N8^i^	0.82 (2)	2.02 (2)	2.810 (5)	159 (4)
O7—H33⋯N5	0.83 (2)	2.09 (2)	2.915 (5)	175 (4)
O8—H35⋯N7^vii^	0.83 (2)	2.03 (2)	2.840 (5)	165 (5)
O8—H36⋯O7	0.83 (2)	2.03 (2)	2.854 (4)	174 (5)
O9—H38⋯N6^vii^	0.84 (2)	2.66 (3)	3.372 (5)	143 (4)
O9—H37⋯O8	0.83 (2)	1.91 (2)	2.715 (5)	161 (5)

Symmetry codes: (i) 
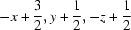
; (ii) 
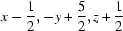
; (iii) 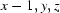; (iv) 
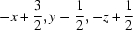
; (v) 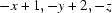; (vi) 
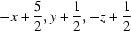
; (vii) 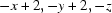.

**Fig. 1.**
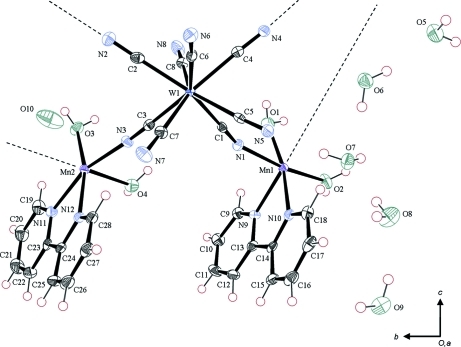
The asymmetric unit in (I). Displacement ellipsoids are drawn at the 50% probability level. The H atoms for the lattice water molecule, O10, were not located in the study.

**Fig. 2.**
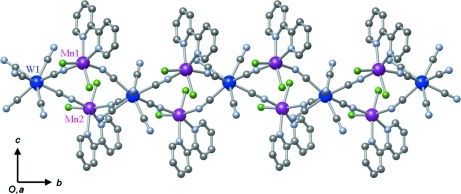
The polymeric structure of (I). Blue, purple, gray, light blue, and green represent W, Mn, C, N and O atoms,respectively. Hydrogen atoms and lattice water molecules are omitted for clarity.

## supplementary crystallographic information

### Comment

In the field of molecular-based magnets, octacyanotungstate [W(CN)_8_]-based magnetic materials are intensively studied due to their functionalities, *e.g.* high Curie temperature (Sieklucka *et al.*, 2009), photo-magnetism (Ohkoshi *et al.*, 2008), and chemically sensitive magnetism (Ohkoshi *et al.*, 2007). Octacyanotungstates of [W(CN)_8_], which can adopt different configurations depending on the coordinating ligands (Leipoldt *et al.*, 1994), also display various structures and dimensionalities. For example, in Mn^II^—W^IV^ cyano-bridged systems, zero-dimensional Mn^II^_4_[W^IV^(CN)_8_]_2_(2,2'-bipyridyl)_8_.14H_2_O (Sieklucka *et al.*, 2000) and three-dimensional Mn^II^_2_[*W*^IV^(CN)_8_](2,2'-bipyrimidine).2H_2_O (Herrera, *et al.*, 2003) have been reported. In this work, we report a one-dimensional cyano-bridged Mn^II^—W^IV^ bimetallic assembly with a bidentate ligand of 2,2'-bipyridyl, [Mn^II^_2_(C_10_H_8_N_2_)_2_(OH_2_)_4_][W^IV^(CN)_8_](C_10_H_8_N_2_)_2_.6H_2_O, (I).

Fig. 1 shows the asymmetric unit of (I). W1 has an eight-coordinated square-anti-prismatic geometry, where four CN groups of [W(CN)_8_] are bridged to Mn1 and Mn2, and the other four CN groups being non-bridging. The coordination geometries of Mn1 and Mn2 are a six-coordinated distorted octahedral. Each Mn atom is coordinated to two nitrogen atoms derived from two CN groups, two nitrogen atoms from 2,2'-bipyridyl, and two oxygen atoms from two water molecules. The asymmetric unit has six zeolitic water molecules. Fig. 2 shows the polymeric structure of (I). The successive connections between W^IV^ and Mn^II^ ions result in the formation of a one-dimensional structure along the *b* axis.

The product of the molar magnetic susceptibility (*χ*_M_) and temperature (*T*), *χ*_M_*T*, at room temperature was determined to be 8.6 cm^3^ K mol^-1^. This value nearly agrees with the expected value of 8.8 cm^3^ K mol^-1^ due to two Mn^II^ (*S* = 5/2, g = 2). The *χ*_M_*T* value gradually decreased below 10 K. This behaviour indicates that the Mn^II^ centres antiferromagnetically interact with each other.

### Experimental

Single crystals of the target compound were prepared by diffusion of aqueous solutions of Mn^II^Cl_2_4H_2_O (3 ml, 0.12 mmol) with 2,2'-bipyridyl (0.13 mmol) and K^I^_4_[W^IV^(CN)_8_]2H_2_O (3 ml, 0.051 mmol) in a test tube. The tube was left in the dark at room temperature, and the yellow platelets grew after several days. Inductively coupled plasma atomic emission spectrometry and CHN elemental analysis confirmed that the formula as (I) less two water molecules. Calculated: C 34.94, H 3.74, N 17.47, Mn 11.43, W 19.13%; found C 34.94, H 3.65, N 17.31, Mn 11.37 W 18.99%. The difference in the number zeolitic water molecules between the elemental analysis and the crystallographic formulation arises as zeolitic water molecules are easy lost from the crystals during the drying process. The CN stretching peaks for Mn^II^—NC—W^IV^ were observed at 2161, 2146, 2134, 2128, 2124, 2110, 2103 and 2081 cm^-1^ in the IR spectrum. In the UV-vis diffuse reflectance spectrum, a broad absorption band was observed with a maximum at 430 nm, which was assigned to the metal-metal charge transfer (MMCT) band of W^IV^—CN—Mn^II^.

### Refinement

The H atoms of the 2,2'-bipyridyl molecules were placed in calculated positions, with C—H = 0.95 Å, and refined using a riding model, with *U*_iso_(H) = 1.2 *U*_eq_(C). The water hydrogen atom positions were refined with O—H distance restraints of 0.84±0.02 Å, and with *U*_iso_(H) = 1.2*U*_eq_(O); the H atoms for the O10 water molecule were not located. The maximum and minimum residual electron density peaks of 2.47 and -1.92 e Å^-3^, respectively, were located 0.89 Å and 0.80 Å from the W1 atom, respectively.

### Crystal data

**Table:**

[Mn_2_W(CN)_8_(C_10_H_8_N_2_)_2_(H_2_O)_4_]·6H_2_O	*F*(000) = 1960
*M**_r_* = 992.40	*D*_x_ = 1.717 Mg m^−^^3^
Monoclinic, *P*2_1_/*n*	Mo *K*α radiation, λ = 0.71075 Å
Hall symbol: -P 2yn	Cell parameters from 28294 reflections
*a* = 11.6673 (3) Å	θ = 3.1–27.5°
*b* = 15.5511 (4) Å	µ = 3.70 mm^−^^1^
*c* = 21.4339 (7) Å	*T* = 90 K
β = 99.213 (1)°	Platelet, yellow
*V* = 3838.78 (18) Å^3^	0.90 × 0.20 × 0.02 mm
*Z* = 4	

### Data collection

**Table:**

Rigaku R-AXIS RAPID diffractometer	8606 independent reflections
Radiation source: fine-focus sealed tube	7342 reflections with *I* > 2σ(*I*)
graphite	*R*_int_ = 0.062
Detector resolution: 10.00 pixels mm^-1^	θ_max_ = 27.5°, θ_min_ = 3.1°
ω scans	*h* = −15→15
Absorption correction: multi-scan (*ABSCOR*; Higashi, 1995)	*k* = −18→19
*T*_min_ = 0.135, *T*_max_ = 0.930	*l* = −27→27
33108 measured reflections	

### Refinement

**Table:**

Refinement on *F*^2^	Primary atom site location: structure-invariant direct methods
Least-squares matrix: full	Secondary atom site location: difference Fourier map
*R*[*F*^2^ > 2σ(*F*^2^)] = 0.036	Hydrogen site location: inferred from neighbouring sites
*wR*(*F*^2^) = 0.079	H atoms treated by a mixture of independent and constrained refinement
*S* = 1.03	*w* = 1/[σ^2^(*F*_o_^2^) + (0.0278*P*)^2^ + 5.0172*P*] where *P* = (*F*_o_^2^ + 2*F*_c_^2^)/3
8606 reflections	(Δ/σ)_max_ = 0.002
533 parameters	Δρ_max_ = 2.47 e Å^−^^3^
18 restraints	Δρ_min_ = −1.92 e Å^−^^3^

### Special details

**Table:**

Geometry. All e.s.d.'s (except the e.s.d. in the dihedral angle between two l.s. planes) are estimated using the full covariance matrix. The cell e.s.d.'s are taken into account individually in the estimation of e.s.d.'s in distances, angles and torsion angles; correlations between e.s.d.'s in cell parameters are only used when they are defined by crystal symmetry. An approximate (isotropic) treatment of cell e.s.d.'s is used for estimating e.s.d.'s involving l.s. planes.Bond lengths and angles W1 - Distance Angles C2 2.1424 (0.0041) C4 2.1437 (0.0038) 107.63 (0.14) C3 2.1494 (0.0039) 76.34 (0.14) 142.60 (0.13) C1 2.1498 (0.0038) 142.96 (0.14) 84.20 (0.13) 73.85 (0.14) C7 2.1591 (0.0039) 84.55 (0.15) 145.08 (0.14) 71.42 (0.14) 105.89 (0.14) C8 2.1700 (0.0040) 73.08 (0.15) 71.85 (0.14) 74.17 (0.14) 78.01 (0.14) 142.45 (0.15) C6 2.1858 (0.0040) 70.17 (0.14) 75.29 (0.14) 136.71 (0.14) 146.11 (0.14) 78.73 (0.14) 119.10 (0.14) C5 2.1872 (0.0040) 143.73 (0.14) 75.60 (0.13) 123.59 (0.13) 72.70 (0.14) 75.84 (0.14) 137.85 (0.14) 76.19 (0.13) W1 - C2 C4 C3 C1 C7 C8 C6 Mn1 - Distance Angles N2_$1 2.1508 (0.0033) N1 2.1762 (0.0033) 96.57 (0.13) O2 2.2209 (0.0030) 82.20 (0.12) 169.50 (0.12) N10 2.2339 (0.0032) 93.95 (0.12) 89.84 (0.12) 100.64 (0.12) O1 2.2440 (0.0029) 92.57 (0.12) 81.90 (0.12) 87.73 (0.12) 170.02 (0.12) N9 2.2542 (0.0029) 159.83 (0.12) 99.14 (0.12) 84.59 (0.11) 73.59 (0.11) 102.08 (0.11) Mn1 - N2_$1 N1 O2 N10 O1 Mn2 - Distance Angles N3 2.1504 (0.0032) N4_$4 2.2012 (0.0032) 92.55 (0.12) O4 2.2194 (0.0030) 83.38 (0.12) 165.86 (0.11) O3 2.2289 (0.0030) 100.21 (0.12) 83.18 (0.11) 84.22 (0.11) N12 2.2482 (0.0030) 93.06 (0.12) 91.00 (0.11) 102.71 (0.11) 165.71 (0.12) N11 2.2493 (0.0032) 162.59 (0.12) 98.25 (0.12) 89.14 (0.11) 94.61 (0.12) 73.24 (0.12) Mn2 - N3 N4_$4 O4 O3 N12 O1 - Distance Angles Mn1 2.2440 (0.0029) H1 0.8363 (0.0190) 108.96 (3.32) H2 0.8417 (0.0190) 110.06 (3.33) 105.52 (4.66) O1 - Mn1 H1 O2 - Distance Angles Mn1 2.2209 (0.0030) H3 0.8337 (0.0190) 132.79 (3.41) H4 0.8222 (0.0191) 120.69 (3.30) 105.61 (4.59) O2 - Mn1 H3 O3 - Distance Angles Mn2 2.2289 (0.0030) H5 0.8240 (0.0190) 109.88 (3.40) H6 0.8286 (0.0191) 118.44 (3.40) 104.60 (4.67) O3 - Mn2 H5 O4 - Distance Angles Mn2 2.2194 (0.0030) H7 0.8366 (0.0189) 120.24 (3.34) H8 0.8296 (0.0189) 114.26 (3.19) 104.47 (4.45) O4 - Mn2 H7 O5 - Distance Angles H29 0.8350 (0.0192) H30 0.8362 (0.0193) 107.18 (4.93) O5 - H29 O6 - Distance Angles H31 0.8332 (0.0191) H32 0.8267 (0.0188) 113.94 (4.49) O6 - H31 O7 - Distance Angles H33 0.8258 (0.0190) H34 0.8236 (0.0189) 104.31 (4.54) O7 - H33 O8 - Distance Angles H36 0.8307 (0.0193) H35 0.8285 (0.0194) 113.61 (5.07) O8 - H36 O9 - Distance Angles H37 0.8344 (0.0189) H38 0.8390 (0.0192) 111.00 (4.58) O9 - H37 N1 - Distance Angles C1 1.1627 (0.0048) Mn1 2.1762 (0.0033) 158.65 (0.31) N1 - C1 N2 - Distance Angles C2 1.1582 (0.0051) Mn1_$4 2.1508 (0.0033) 159.78 (0.31) N2 - C2 N3 - Distance Angles C3 1.1545 (0.0048) Mn2 2.1504 (0.0032) 176.05 (0.31) N3 - C3 N4 - Distance Angles C4 1.1529 (0.0047) Mn2_$1 2.2011 (0.0032) 146.40 (0.30) N4 - C4 N5 - Distance Angles C5 1.1465 (0.0049) N5 - N6 - Distance Angles C6 1.1526 (0.0051) N6 - N7 - Distance Angles C7 1.1597 (0.0050) N7 - N8 - Distance Angles C8 1.1575 (0.0052) N8 - N9 - Distance Angles C9 1.3429 (0.0050) C13 1.3559 (0.0045) 118.44 (0.31) Mn1 2.2542 (0.0029) 124.73 (0.24) 116.59 (0.24) N9 - C9 C13 N10 - Distance Angles C18 1.3462 (0.0050) C14 1.3514 (0.0048) 118.82 (0.33) Mn1 2.2339 (0.0032) 123.80 (0.25) 117.30 (0.25) N10 - C18 C14 N11 - Distance Angles C19 1.3327 (0.0053) C23 1.3506 (0.0048) 119.05 (0.34) Mn2 2.2493 (0.0032) 124.16 (0.27) 116.66 (0.26) N11 - C19 C23 N12 - Distance Angles C28 1.3420 (0.0050) C24 1.3487 (0.0048) 118.53 (0.32) Mn2 2.2482 (0.0030) 124.07 (0.25) 117.37 (0.25) N12 - C28 C24 C1 - Distance Angles N1 1.1627 (0.0048) W1 2.1498 (0.0038) 178.55 (0.34) C1 - N1 C2 - Distance Angles N2 1.1582 (0.0051) W1 2.1424 (0.0041) 177.68 (0.34) C2 - N2 C3 - Distance Angles N3 1.1545 (0.0048) W1 2.1494 (0.0039) 178.65 (0.34) C3 - N3 C4 - Distance Angles N4 1.1529 (0.0047) W1 2.1437 (0.0038) 175.63 (0.33) C4 - N4 C5 - Distance Angles N5 1.1465 (0.0049) W1 2.1872 (0.0039) 176.70 (0.31) C5 - N5 C6 - Distance Angles N6 1.1526 (0.0051) W1 2.1858 (0.0040) 177.03 (0.32) C6 - N6 C7 - Distance Angles N7 1.1597 (0.0050) W1 2.1591 (0.0039) 178.24 (0.37) C7 - N7 C8 - Distance Angles N8 1.1575 (0.0052) W1 2.1700 (0.0040) 178.82 (0.38) C8 - N8 C9 - Distance Angles N9 1.3429 (0.0050) C10 1.3907 (0.0053) 123.11 (0.36) H9 0.9500 118.44 118.44 C9 - N9 C10 C10 - Distance Angles C11 1.3817 (0.0057) C9 1.3907 (0.0053) 117.87 (0.37) H10 0.9500 121.07 121.07 C10 - C11 C9 C11 - Distance Angles C10 1.3817 (0.0057) C12 1.3945 (0.0060) 119.57 (0.36) H11 0.9500 120.22 120.22 C11 - C10 C12 C12 - Distance Angles C13 1.3781 (0.0052) C11 1.3945 (0.0060) 119.19 (0.36) H12 0.9500 120.41 120.41 C12 - C13 C11 C13 - Distance Angles N9 1.3559 (0.0045) C12 1.3781 (0.0052) 121.72 (0.36) C14 1.4931 (0.0055) 115.99 (0.32) 122.28 (0.35) C13 - N9 C12 C14 - Distance Angles N10 1.3514 (0.0048) C15 1.3977 (0.0051) 121.38 (0.36) C13 1.4931 (0.0054) 116.37 (0.32) 122.22 (0.35) C14 - N10 C15 C15 - Distance Angles C16 1.3876 (0.0061) C14 1.3977 (0.0051) 119.00 (0.38) H15 0.9500 120.50 120.50 C15 - C16 C14 C16 - Distance Angles C17 1.3835 (0.0060) C15 1.3876 (0.0061) 119.36 (0.37) H16 0.9500 120.32 120.32 C16 - C17 C15 C17 - Distance Angles C18 1.3809 (0.0054) C16 1.3835 (0.0060) 118.75 (0.39) H17 0.9500 120.63 120.63 C17 - C18 C16 C18 - Distance Angles N10 1.3462 (0.0049) C17 1.3809 (0.0054) 122.69 (0.37) H18 0.9500 118.66 118.66 C18 - N10 C17 C19 - Distance Angles N11 1.3327 (0.0053) C20 1.3846 (0.0058) 122.88 (0.42) H19 0.9500 118.56 118.56 C19 - N11 C20 C20 - Distance Angles C21 1.3833 (0.0064) C19 1.3846 (0.0058) 118.35 (0.44) H20 0.9500 120.82 120.82 C20 - C21 C19 C21 - Distance Angles C22 1.3743 (0.0066) C20 1.3833 (0.0064) 119.29 (0.40) H21 0.9500 120.36 120.36 C21 - C22 C20 C22 - Distance Angles C21 1.3743 (0.0066) C23 1.3955 (0.0055) 119.60 (0.41) H22 0.9500 120.20 120.20 C22 - C21 C23 C23 - Distance Angles N11 1.3506 (0.0048) C22 1.3955 (0.0055) 120.82 (0.38) C24 1.4917 (0.0058) 116.51 (0.33) 122.62 (0.37) C23 - N11 C22 C24 - Distance Angles N12 1.3487 (0.0048) C25 1.3938 (0.0054) 121.71 (0.39) C23 1.4917 (0.0058) 115.76 (0.33) 122.50 (0.37) C24 - N12 C25 C25 - Distance Angles C26 1.3784 (0.0067) C24 1.3938 (0.0054) 118.98 (0.40) H25 0.9500 120.51 120.51 C25 - C26 C24 C26 - Distance Angles C25 1.3784 (0.0067) C27 1.3910 (0.0061) 119.72 (0.39) H26 0.9500 120.14 120.14 C26 - C25 C27 C27 - Distance Angles C28 1.3840 (0.0053) C26 1.3910 (0.0061) 117.90 (0.40) H27 0.9500 121.05 121.05 C27 - C28 C26 C28 - Distance Angles N12 1.3420 (0.0050) C27 1.3840 (0.0053) 123.16 (0.37) H28 0.9500 118.42 118.42 C28 - N12 C27
Refinement. Refinement of *F*^2^ against ALL reflections. The weighted *R*-factor *wR* and goodness of fit *S* are based on *F*^2^, conventional *R*-factors *R* are based on *F*, with *F* set to zero for negative *F*^2^. The threshold expression of *F*^2^ > σ(*F*^2^) is used only for calculating *R*-factors(gt) *etc*. and is not relevant to the choice of reflections for refinement. *R*-factors based on *F*^2^ are statistically about twice as large as those based on *F*, and *R*- factors based on ALL data will be even larger.

### Fractional atomic coordinates and isotropic or equivalent isotropic displacement parameters (Å^2^)

**Table:**

	*x*	*y*	*z*	*U*_iso_*/*U*_eq_	
W1	0.810420 (12)	0.888675 (9)	0.224158 (6)	0.01102 (6)	
Mn1	0.45358 (5)	1.11286 (4)	0.17557 (3)	0.01337 (12)	
Mn2	0.47767 (5)	0.64730 (4)	0.15037 (2)	0.01383 (12)	
O1	0.4226 (3)	1.0759 (2)	0.27269 (13)	0.0256 (7)	
H1	0.487 (2)	1.069 (3)	0.2960 (19)	0.031*	
H2	0.390 (4)	1.116 (2)	0.289 (2)	0.031*	
O2	0.3345 (2)	1.2232 (2)	0.18036 (15)	0.0263 (7)	
H3	0.2623 (17)	1.227 (3)	0.175 (2)	0.032*	
H4	0.359 (4)	1.2694 (18)	0.196 (2)	0.032*	
O3	0.4296 (3)	0.6143 (2)	0.24405 (14)	0.0256 (7)	
H5	0.482 (3)	0.586 (3)	0.265 (2)	0.031*	
H6	0.417 (4)	0.654 (2)	0.2679 (18)	0.031*	
O4	0.3504 (2)	0.7532 (2)	0.15113 (13)	0.0233 (6)	
H7	0.281 (2)	0.742 (3)	0.154 (2)	0.028*	
H8	0.370 (4)	0.790 (2)	0.1789 (17)	0.028*	
O5	1.1463 (3)	1.3841 (2)	0.25177 (16)	0.0298 (7)	
H29	1.110 (4)	1.4308 (19)	0.249 (2)	0.036*	
H30	1.217 (2)	1.396 (3)	0.253 (2)	0.036*	
O6	1.1027 (3)	1.24366 (19)	0.17341 (14)	0.0254 (7)	
H31	1.096 (4)	1.288 (2)	0.1945 (19)	0.030*	
H32	1.055 (3)	1.240 (3)	0.1406 (14)	0.030*	
O7	0.9086 (3)	1.22249 (19)	0.08148 (14)	0.0233 (6)	
H33	0.898 (4)	1.1743 (17)	0.096 (2)	0.028*	
H34	0.866 (3)	1.254 (3)	0.0984 (19)	0.028*	
O8	0.9834 (3)	1.2948 (2)	−0.02763 (16)	0.0358 (8)	
H36	0.956 (4)	1.274 (3)	0.0026 (17)	0.043*	
H35	1.035 (3)	1.265 (3)	−0.039 (2)	0.043*	
O9	0.8777 (3)	1.2844 (2)	−0.14985 (14)	0.0283 (7)	
H37	0.901 (4)	1.298 (3)	−0.1123 (11)	0.034*	
H38	0.913 (4)	1.241 (2)	−0.160 (2)	0.034*	
O10	0.8624 (3)	0.5937 (3)	0.14671 (17)	0.0539 (11)	
N1	0.5679 (3)	1.0016 (2)	0.18906 (15)	0.0190 (7)	
N2	0.9176 (3)	0.7068 (2)	0.28805 (16)	0.0213 (7)	
N3	0.6026 (3)	0.7475 (2)	0.17782 (14)	0.0175 (7)	
N4	0.8920 (3)	1.0454 (2)	0.32493 (14)	0.0178 (7)	
N5	0.8726 (3)	1.0573 (2)	0.13964 (15)	0.0204 (7)	
N6	1.1000 (3)	0.8869 (2)	0.24367 (16)	0.0223 (8)	
N7	0.8601 (3)	0.7988 (2)	0.09063 (16)	0.0277 (8)	
N8	0.6807 (3)	0.8526 (2)	0.34906 (16)	0.0286 (8)	
N9	0.3003 (3)	1.0525 (2)	0.11361 (13)	0.0148 (7)	
N10	0.4871 (3)	1.1251 (2)	0.07622 (15)	0.0166 (7)	
N11	0.3369 (3)	0.5667 (2)	0.09489 (15)	0.0195 (7)	
N12	0.5095 (3)	0.6474 (2)	0.04966 (14)	0.0155 (7)	
C1	0.6537 (3)	0.9626 (2)	0.20079 (16)	0.0144 (8)	
C2	0.8776 (3)	0.7701 (3)	0.26592 (17)	0.0186 (8)	
C3	0.6743 (3)	0.7977 (3)	0.19365 (16)	0.0149 (8)	
C4	0.8609 (3)	0.9891 (2)	0.29147 (16)	0.0142 (8)	
C5	0.8501 (3)	0.9979 (3)	0.16697 (16)	0.0145 (8)	
C6	1.0001 (3)	0.8900 (2)	0.23681 (17)	0.0163 (8)	
C7	0.8414 (3)	0.8311 (3)	0.13671 (17)	0.0180 (8)	
C8	0.7253 (3)	0.8661 (3)	0.30556 (18)	0.0189 (8)	
C9	0.2107 (3)	1.0146 (3)	0.13495 (18)	0.0203 (8)	
H9	0.2054	1.0196	0.1786	0.024*	
C10	0.1254 (3)	0.9682 (3)	0.09624 (19)	0.0234 (9)	
H10	0.0647	0.9403	0.1131	0.028*	
C11	0.1318 (3)	0.9641 (3)	0.03249 (19)	0.0258 (10)	
H11	0.0754	0.9326	0.0047	0.031*	
C12	0.2215 (3)	1.0065 (3)	0.00926 (18)	0.0225 (9)	
H12	0.2247	1.0065	−0.0347	0.027*	
C13	0.3053 (3)	1.0485 (3)	0.05091 (17)	0.0172 (8)	
C14	0.4080 (3)	1.0904 (2)	0.03018 (17)	0.0156 (8)	
C15	0.4242 (4)	1.0915 (3)	−0.03304 (18)	0.0210 (9)	
H15	0.3675	1.0673	−0.0650	0.025*	
C16	0.5243 (4)	1.1284 (3)	−0.04842 (19)	0.0257 (10)	
H16	0.5369	1.1297	−0.0911	0.031*	
C17	0.6054 (4)	1.1632 (3)	−0.00101 (19)	0.0244 (9)	
H17	0.6749	1.1882	−0.0105	0.029*	
C18	0.5835 (3)	1.1609 (3)	0.06040 (18)	0.0190 (8)	
H18	0.6387	1.1857	0.0928	0.023*	
C19	0.2547 (4)	0.5260 (3)	0.1201 (2)	0.0264 (9)	
H19	0.2584	0.5274	0.1647	0.032*	
C20	0.1643 (4)	0.4819 (3)	0.0842 (2)	0.0325 (11)	
H20	0.1072	0.4534	0.1036	0.039*	
C21	0.1592 (4)	0.4803 (3)	0.0193 (2)	0.0335 (11)	
H21	0.0981	0.4507	−0.0067	0.040*	
C22	0.2434 (4)	0.5220 (3)	−0.0073 (2)	0.0301 (10)	
H22	0.2405	0.5219	−0.0518	0.036*	
C23	0.3331 (3)	0.5645 (3)	0.03160 (18)	0.0193 (8)	
C24	0.4317 (4)	0.6061 (2)	0.00663 (18)	0.0183 (8)	
C25	0.4452 (4)	0.6007 (3)	−0.05668 (19)	0.0270 (10)	
H25	0.3892	0.5717	−0.0864	0.032*	
C26	0.5413 (4)	0.6380 (3)	−0.0755 (2)	0.0296 (10)	
H26	0.5519	0.6350	−0.1185	0.036*	
C27	0.6226 (4)	0.6800 (3)	−0.03138 (18)	0.0238 (9)	
H27	0.6898	0.7055	−0.0433	0.029*	
C28	0.6025 (3)	0.6833 (3)	0.03048 (18)	0.0195 (8)	
H28	0.6571	0.7125	0.0608	0.023*	

### Atomic displacement parameters (Å^2^)

**Table:**

	*U*^11^	*U*^22^	*U*^33^	*U*^12^	*U*^13^	*U*^23^
W1	0.01106 (9)	0.01089 (9)	0.01059 (8)	0.00028 (6)	0.00020 (5)	−0.00007 (5)
Mn1	0.0120 (3)	0.0139 (3)	0.0135 (3)	0.0009 (2)	0.0000 (2)	−0.0007 (2)
Mn2	0.0134 (3)	0.0148 (3)	0.0129 (3)	−0.0006 (2)	0.0007 (2)	−0.0002 (2)
O1	0.0334 (18)	0.0266 (17)	0.0183 (15)	0.0010 (15)	0.0087 (12)	−0.0014 (12)
O2	0.0149 (14)	0.0209 (17)	0.0412 (18)	0.0019 (13)	−0.0012 (13)	−0.0107 (13)
O3	0.0317 (17)	0.0262 (18)	0.0205 (15)	0.0088 (14)	0.0095 (13)	0.0049 (12)
O4	0.0155 (14)	0.0284 (18)	0.0263 (16)	0.0017 (14)	0.0045 (12)	−0.0002 (12)
O5	0.0197 (16)	0.0264 (18)	0.0425 (19)	0.0022 (14)	0.0022 (14)	−0.0066 (14)
O6	0.0232 (16)	0.0232 (17)	0.0286 (17)	0.0015 (14)	0.0009 (12)	−0.0042 (13)
O7	0.0291 (16)	0.0187 (16)	0.0238 (15)	0.0002 (14)	0.0098 (12)	0.0006 (12)
O8	0.0362 (19)	0.043 (2)	0.0322 (18)	0.0063 (17)	0.0168 (15)	0.0078 (15)
O9	0.0208 (15)	0.036 (2)	0.0292 (16)	0.0044 (14)	0.0079 (13)	0.0056 (14)
O10	0.043 (2)	0.082 (3)	0.037 (2)	−0.024 (2)	0.0055 (17)	−0.0118 (19)
N1	0.0197 (17)	0.0174 (17)	0.0192 (16)	0.0025 (15)	0.0011 (13)	−0.0010 (13)
N2	0.0149 (16)	0.0215 (19)	0.0274 (18)	0.0006 (15)	0.0031 (13)	0.0037 (15)
N3	0.0150 (16)	0.0187 (18)	0.0184 (16)	−0.0007 (15)	0.0012 (12)	−0.0032 (13)
N4	0.0216 (17)	0.0176 (18)	0.0139 (16)	−0.0047 (15)	0.0016 (13)	0.0024 (13)
N5	0.0264 (18)	0.0180 (18)	0.0165 (16)	−0.0043 (15)	0.0024 (13)	0.0007 (13)
N6	0.0191 (19)	0.023 (2)	0.0252 (18)	−0.0017 (15)	0.0042 (14)	0.0036 (14)
N7	0.0290 (19)	0.031 (2)	0.0245 (19)	−0.0028 (17)	0.0078 (15)	−0.0086 (16)
N8	0.038 (2)	0.025 (2)	0.0241 (19)	−0.0143 (18)	0.0110 (16)	−0.0029 (15)
N9	0.0128 (15)	0.0194 (18)	0.0120 (15)	0.0008 (14)	0.0014 (12)	−0.0004 (12)
N10	0.0192 (17)	0.0133 (17)	0.0175 (16)	0.0021 (14)	0.0034 (13)	0.0017 (12)
N11	0.0170 (16)	0.0186 (18)	0.0217 (17)	0.0005 (15)	−0.0010 (13)	0.0002 (14)
N12	0.0174 (16)	0.0151 (17)	0.0143 (15)	0.0025 (14)	0.0028 (12)	0.0010 (12)
C1	0.0200 (19)	0.0131 (19)	0.0106 (17)	−0.0020 (17)	0.0040 (14)	−0.0030 (13)
C2	0.0145 (19)	0.024 (2)	0.0165 (19)	−0.0045 (17)	0.0011 (14)	0.0007 (16)
C3	0.0152 (18)	0.016 (2)	0.0144 (17)	0.0056 (17)	0.0052 (14)	0.0012 (14)
C4	0.0108 (17)	0.018 (2)	0.0143 (18)	0.0011 (16)	0.0039 (13)	0.0022 (15)
C5	0.0118 (18)	0.018 (2)	0.0132 (17)	−0.0020 (16)	0.0003 (13)	−0.0056 (15)
C6	0.021 (2)	0.0124 (19)	0.0155 (18)	0.0018 (16)	0.0038 (15)	0.0011 (14)
C7	0.0161 (19)	0.019 (2)	0.0187 (19)	−0.0046 (17)	0.0029 (15)	0.0002 (16)
C8	0.022 (2)	0.018 (2)	0.0163 (19)	−0.0001 (17)	−0.0004 (16)	−0.0009 (15)
C9	0.020 (2)	0.021 (2)	0.0195 (19)	0.0019 (18)	0.0037 (15)	0.0003 (16)
C10	0.0148 (19)	0.027 (2)	0.028 (2)	−0.0047 (18)	0.0024 (16)	0.0007 (17)
C11	0.019 (2)	0.030 (2)	0.026 (2)	0.0003 (19)	−0.0062 (16)	−0.0091 (18)
C12	0.025 (2)	0.025 (2)	0.0152 (19)	0.0021 (19)	−0.0021 (16)	−0.0027 (16)
C13	0.0168 (19)	0.017 (2)	0.0175 (18)	0.0056 (17)	−0.0001 (15)	0.0015 (15)
C14	0.0177 (19)	0.0133 (19)	0.0163 (18)	0.0072 (16)	0.0041 (14)	0.0046 (14)
C15	0.028 (2)	0.020 (2)	0.0142 (18)	0.0077 (18)	0.0037 (16)	0.0024 (15)
C16	0.034 (2)	0.025 (2)	0.020 (2)	0.011 (2)	0.0123 (18)	0.0064 (17)
C17	0.025 (2)	0.022 (2)	0.028 (2)	0.0046 (19)	0.0121 (17)	0.0100 (17)
C18	0.0126 (18)	0.018 (2)	0.027 (2)	0.0014 (17)	0.0049 (15)	0.0027 (16)
C19	0.022 (2)	0.025 (2)	0.033 (2)	−0.0023 (19)	0.0067 (18)	−0.0006 (18)
C20	0.022 (2)	0.027 (3)	0.047 (3)	−0.005 (2)	0.003 (2)	−0.002 (2)
C21	0.024 (2)	0.029 (3)	0.043 (3)	−0.005 (2)	−0.006 (2)	−0.007 (2)
C22	0.033 (2)	0.027 (2)	0.025 (2)	0.005 (2)	−0.0101 (19)	−0.0022 (18)
C23	0.022 (2)	0.012 (2)	0.022 (2)	0.0077 (17)	−0.0013 (16)	−0.0004 (15)
C24	0.023 (2)	0.015 (2)	0.0157 (18)	0.0065 (17)	0.0001 (15)	0.0016 (14)
C25	0.038 (3)	0.028 (2)	0.0140 (19)	0.007 (2)	−0.0021 (17)	−0.0022 (16)
C26	0.046 (3)	0.026 (2)	0.018 (2)	0.013 (2)	0.0090 (19)	0.0028 (17)
C27	0.031 (2)	0.021 (2)	0.021 (2)	0.0073 (19)	0.0110 (17)	0.0046 (16)
C28	0.023 (2)	0.017 (2)	0.0181 (19)	0.0040 (18)	0.0033 (15)	0.0017 (15)

### Geometric parameters (Å, °)

**Table:**

W1—C2	2.142 (4)	N6—C6	1.153 (5)
W1—C4	2.144 (4)	N7—C7	1.160 (5)
W1—C3	2.149 (4)	N8—C8	1.157 (5)
W1—C1	2.150 (4)	N9—C9	1.343 (5)
W1—C7	2.159 (4)	N9—C13	1.356 (5)
W1—C8	2.170 (4)	N10—C18	1.346 (5)
W1—C6	2.186 (4)	N10—C14	1.351 (5)
W1—C5	2.187 (4)	N11—C19	1.333 (5)
Mn1—N2^i^	2.151 (3)	N11—C23	1.351 (5)
Mn1—N1	2.176 (3)	N12—C28	1.342 (5)
Mn1—O2	2.221 (3)	N12—C24	1.349 (5)
Mn1—N10	2.234 (3)	C9—C10	1.391 (5)
Mn1—O1	2.244 (3)	C9—H9	0.9500
Mn1—N9	2.254 (3)	C10—C11	1.382 (6)
Mn2—N3	2.150 (3)	C10—H10	0.9500
Mn2—N4^ii^	2.201 (3)	C11—C12	1.395 (6)
Mn2—O4	2.219 (3)	C11—H11	0.9500
Mn2—O3	2.229 (3)	C12—C13	1.378 (5)
Mn2—N12	2.248 (3)	C12—H12	0.9500
Mn2—N11	2.249 (3)	C13—C14	1.493 (5)
O1—H1	0.836 (19)	C14—C15	1.398 (5)
O1—H2	0.842 (19)	C15—C16	1.388 (6)
O2—H3	0.834 (19)	C15—H15	0.9500
O2—H4	0.822 (19)	C16—C17	1.384 (6)
O3—H5	0.824 (19)	C16—H16	0.9500
O3—H6	0.829 (19)	C17—C18	1.381 (5)
O4—H7	0.837 (19)	C17—H17	0.9500
O4—H8	0.830 (19)	C18—H18	0.9500
O5—H29	0.835 (19)	C19—C20	1.385 (6)
O5—H30	0.836 (19)	C19—H19	0.9500
O6—H31	0.833 (19)	C20—C21	1.383 (6)
O6—H32	0.827 (19)	C20—H20	0.9500
O7—H33	0.826 (19)	C21—C22	1.374 (7)
O7—H34	0.824 (19)	C21—H21	0.9500
O8—H36	0.831 (19)	C22—C23	1.396 (5)
O8—H35	0.828 (19)	C22—H22	0.9500
O9—H37	0.834 (19)	C23—C24	1.492 (6)
O9—H38	0.839 (19)	C24—C25	1.394 (5)
N1—C1	1.163 (5)	C25—C26	1.378 (7)
N2—C2	1.158 (5)	C25—H25	0.9500
N2—Mn1^ii^	2.151 (3)	C26—C27	1.391 (6)
N3—C3	1.154 (5)	C26—H26	0.9500
N4—C4	1.153 (5)	C27—C28	1.384 (5)
N4—Mn2^i^	2.201 (3)	C27—H27	0.9500
N5—C5	1.146 (5)	C28—H28	0.9500
			
C2—W1—C4	107.63 (14)	C9—N9—Mn1	124.7 (2)
C2—W1—C3	76.34 (14)	C13—N9—Mn1	116.6 (2)
C4—W1—C3	142.60 (13)	C18—N10—C14	118.8 (3)
C2—W1—C1	142.96 (14)	C18—N10—Mn1	123.8 (3)
C4—W1—C1	84.20 (13)	C14—N10—Mn1	117.3 (2)
C3—W1—C1	73.85 (14)	C19—N11—C23	119.1 (3)
C2—W1—C7	84.55 (15)	C19—N11—Mn2	124.2 (3)
C4—W1—C7	145.08 (14)	C23—N11—Mn2	116.7 (3)
C3—W1—C7	71.42 (14)	C28—N12—C24	118.5 (3)
C1—W1—C7	105.89 (14)	C28—N12—Mn2	124.1 (2)
C2—W1—C8	73.08 (15)	C24—N12—Mn2	117.4 (3)
C4—W1—C8	71.85 (14)	N1—C1—W1	178.5 (3)
C3—W1—C8	74.17 (14)	N2—C2—W1	177.7 (3)
C1—W1—C8	78.01 (14)	N3—C3—W1	178.6 (3)
C7—W1—C8	142.45 (15)	N4—C4—W1	175.6 (3)
C2—W1—C6	70.17 (14)	N5—C5—W1	176.7 (3)
C4—W1—C6	75.29 (14)	N6—C6—W1	177.0 (3)
C3—W1—C6	136.71 (14)	N7—C7—W1	178.2 (4)
C1—W1—C6	146.11 (14)	N8—C8—W1	178.8 (4)
C7—W1—C6	78.73 (14)	N9—C9—C10	123.1 (4)
C8—W1—C6	119.10 (14)	N9—C9—H9	118.4
C2—W1—C5	143.73 (14)	C10—C9—H9	118.4
C4—W1—C5	75.60 (13)	C11—C10—C9	117.9 (4)
C3—W1—C5	123.59 (13)	C11—C10—H10	121.1
C1—W1—C5	72.70 (14)	C9—C10—H10	121.1
C7—W1—C5	75.84 (14)	C10—C11—C12	119.6 (4)
C8—W1—C5	137.85 (14)	C10—C11—H11	120.2
C6—W1—C5	76.19 (13)	C12—C11—H11	120.2
N2^i^—Mn1—N1	96.57 (13)	C13—C12—C11	119.2 (4)
N2^i^—Mn1—O2	82.20 (12)	C13—C12—H12	120.4
N1—Mn1—O2	169.50 (12)	C11—C12—H12	120.4
N2^i^—Mn1—N10	93.95 (12)	N9—C13—C12	121.7 (4)
N1—Mn1—N10	89.84 (12)	N9—C13—C14	116.0 (3)
O2—Mn1—N10	100.64 (12)	C12—C13—C14	122.3 (3)
N2^i^—Mn1—O1	92.57 (12)	N10—C14—C15	121.4 (4)
N1—Mn1—O1	81.90 (12)	N10—C14—C13	116.4 (3)
O2—Mn1—O1	87.73 (12)	C15—C14—C13	122.2 (4)
N10—Mn1—O1	170.02 (12)	C16—C15—C14	119.0 (4)
N2^i^—Mn1—N9	159.83 (12)	C16—C15—H15	120.5
N1—Mn1—N9	99.14 (12)	C14—C15—H15	120.5
O2—Mn1—N9	84.59 (11)	C17—C16—C15	119.4 (4)
N10—Mn1—N9	73.59 (11)	C17—C16—H16	120.3
O1—Mn1—N9	102.08 (11)	C15—C16—H16	120.3
N3—Mn2—N4^ii^	92.55 (12)	C18—C17—C16	118.7 (4)
N3—Mn2—O4	83.38 (12)	C18—C17—H17	120.6
N4^ii^—Mn2—O4	165.86 (11)	C16—C17—H17	120.6
N3—Mn2—O3	100.21 (12)	N10—C18—C17	122.7 (4)
N4^ii^—Mn2—O3	83.18 (11)	N10—C18—H18	118.7
O4—Mn2—O3	84.22 (11)	C17—C18—H18	118.7
N3—Mn2—N12	93.06 (12)	N11—C19—C20	122.9 (4)
N4^ii^—Mn2—N12	91.00 (11)	N11—C19—H19	118.6
O4—Mn2—N12	102.71 (11)	C20—C19—H19	118.6
O3—Mn2—N12	165.71 (12)	C21—C20—C19	118.4 (4)
N3—Mn2—N11	162.59 (12)	C21—C20—H20	120.8
N4^ii^—Mn2—N11	98.25 (12)	C19—C20—H20	120.8
O4—Mn2—N11	89.14 (11)	C22—C21—C20	119.3 (4)
O3—Mn2—N11	94.61 (12)	C22—C21—H21	120.4
N12—Mn2—N11	73.24 (12)	C20—C21—H21	120.4
Mn1—O1—H1	109 (3)	C21—C22—C23	119.6 (4)
Mn1—O1—H2	110 (3)	C21—C22—H22	120.2
H1—O1—H2	106 (5)	C23—C22—H22	120.2
Mn1—O2—H3	133 (3)	N11—C23—C22	120.8 (4)
Mn1—O2—H4	121 (3)	N11—C23—C24	116.5 (3)
H3—O2—H4	106 (5)	C22—C23—C24	122.6 (4)
Mn2—O3—H5	110 (3)	N12—C24—C25	121.7 (4)
Mn2—O3—H6	118 (3)	N12—C24—C23	115.8 (3)
H5—O3—H6	105 (5)	C25—C24—C23	122.5 (4)
Mn2—O4—H7	120 (3)	C26—C25—C24	119.0 (4)
Mn2—O4—H8	114 (3)	C26—C25—H25	120.5
H7—O4—H8	104 (4)	C24—C25—H25	120.5
H29—O5—H30	107 (5)	C25—C26—C27	119.7 (4)
H31—O6—H32	114 (4)	C25—C26—H26	120.1
H33—O7—H34	104 (5)	C27—C26—H26	120.1
H36—O8—H35	114 (5)	C28—C27—C26	117.9 (4)
H37—O9—H38	111 (5)	C28—C27—H27	121.0
C1—N1—Mn1	158.7 (3)	C26—C27—H27	121.0
C2—N2—Mn1^ii^	159.8 (3)	N12—C28—C27	123.2 (4)
C3—N3—Mn2	176.1 (3)	N12—C28—H28	118.4
C4—N4—Mn2^i^	146.4 (3)	C27—C28—H28	118.4
C9—N9—C13	118.4 (3)		
			
N2^i^—Mn1—N1—C1	−7.6 (8)	C5—W1—C4—N4	−25 (4)
O2—Mn1—N1—C1	−90.2 (11)	C2—W1—C5—N5	−95 (6)
N10—Mn1—N1—C1	86.4 (8)	C4—W1—C5—N5	5(6)
O1—Mn1—N1—C1	−99.3 (8)	C3—W1—C5—N5	149 (6)
N9—Mn1—N1—C1	159.7 (8)	C1—W1—C5—N5	93 (6)
N4^ii^—Mn2—N3—C3	−3(4)	C7—W1—C5—N5	−155 (6)
O4—Mn2—N3—C3	163 (4)	C8—W1—C5—N5	45 (6)
O3—Mn2—N3—C3	80 (4)	C6—W1—C5—N5	−73 (6)
N12—Mn2—N3—C3	−94 (4)	C2—W1—C6—N6	19 (6)
N11—Mn2—N3—C3	−132 (4)	C4—W1—C6—N6	135 (6)
N2^i^—Mn1—N9—C9	−128.7 (4)	C3—W1—C6—N6	−22 (7)
N1—Mn1—N9—C9	90.6 (3)	C1—W1—C6—N6	−171 (6)
O2—Mn1—N9—C9	−79.5 (3)	C7—W1—C6—N6	−69 (6)
N10—Mn1—N9—C9	177.7 (3)	C8—W1—C6—N6	76 (6)
O1—Mn1—N9—C9	7.0 (3)	C5—W1—C6—N6	−147 (6)
N2^i^—Mn1—N9—C13	57.1 (5)	C2—W1—C7—N7	−20 (12)
N1—Mn1—N9—C13	−83.6 (3)	C4—W1—C7—N7	94 (12)
O2—Mn1—N9—C13	106.3 (3)	C3—W1—C7—N7	−97 (12)
N10—Mn1—N9—C13	3.6 (3)	C1—W1—C7—N7	−163 (12)
O1—Mn1—N9—C13	−167.2 (3)	C8—W1—C7—N7	−72 (12)
N2^i^—Mn1—N10—C18	17.3 (3)	C6—W1—C7—N7	51 (12)
N1—Mn1—N10—C18	−79.3 (3)	C5—W1—C7—N7	130 (12)
O2—Mn1—N10—C18	100.1 (3)	C2—W1—C8—N8	−11 (18)
O1—Mn1—N10—C18	−113.3 (7)	C4—W1—C8—N8	−127 (18)
N9—Mn1—N10—C18	−178.8 (3)	C3—W1—C8—N8	69 (18)
N2^i^—Mn1—N10—C14	−166.1 (3)	C1—W1—C8—N8	145 (18)
N1—Mn1—N10—C14	97.3 (3)	C7—W1—C8—N8	45 (18)
O2—Mn1—N10—C14	−83.3 (3)	C6—W1—C8—N8	−66 (18)
O1—Mn1—N10—C14	63.3 (8)	C5—W1—C8—N8	−168 (18)
N9—Mn1—N10—C14	−2.2 (3)	C13—N9—C9—C10	2.8 (6)
N3—Mn2—N11—C19	−142.6 (4)	Mn1—N9—C9—C10	−171.3 (3)
N4^ii^—Mn2—N11—C19	89.6 (3)	N9—C9—C10—C11	−2.4 (6)
O4—Mn2—N11—C19	−78.3 (3)	C9—C10—C11—C12	−0.6 (6)
O3—Mn2—N11—C19	5.8 (3)	C10—C11—C12—C13	3.0 (6)
N12—Mn2—N11—C19	178.1 (4)	C9—N9—C13—C12	−0.2 (6)
N3—Mn2—N11—C23	33.3 (6)	Mn1—N9—C13—C12	174.4 (3)
N4^ii^—Mn2—N11—C23	−94.5 (3)	C9—N9—C13—C14	−178.9 (3)
O4—Mn2—N11—C23	97.6 (3)	Mn1—N9—C13—C14	−4.3 (4)
O3—Mn2—N11—C23	−178.2 (3)	C11—C12—C13—N9	−2.7 (6)
N12—Mn2—N11—C23	−5.9 (3)	C11—C12—C13—C14	176.0 (4)
N3—Mn2—N12—C28	16.4 (3)	C18—N10—C14—C15	−0.3 (5)
N4^ii^—Mn2—N12—C28	−76.2 (3)	Mn1—N10—C14—C15	−177.1 (3)
O4—Mn2—N12—C28	100.3 (3)	C18—N10—C14—C13	177.6 (3)
O3—Mn2—N12—C28	−141.9 (4)	Mn1—N10—C14—C13	0.8 (4)
N11—Mn2—N12—C28	−174.5 (3)	N9—C13—C14—N10	2.4 (5)
N3—Mn2—N12—C24	−165.9 (3)	C12—C13—C14—N10	−176.4 (4)
N4^ii^—Mn2—N12—C24	101.5 (3)	N9—C13—C14—C15	−179.8 (3)
O4—Mn2—N12—C24	−82.0 (3)	C12—C13—C14—C15	1.5 (6)
O3—Mn2—N12—C24	35.8 (6)	N10—C14—C15—C16	0.6 (6)
N11—Mn2—N12—C24	3.2 (3)	C13—C14—C15—C16	−177.2 (4)
Mn1—N1—C1—W1	127 (14)	C14—C15—C16—C17	0.0 (6)
C2—W1—C1—N1	19 (14)	C15—C16—C17—C18	−0.7 (6)
C4—W1—C1—N1	−93 (14)	C14—N10—C18—C17	−0.5 (6)
C3—W1—C1—N1	56 (14)	Mn1—N10—C18—C17	176.1 (3)
C7—W1—C1—N1	121 (14)	C16—C17—C18—N10	1.0 (6)
C8—W1—C1—N1	−20 (14)	C23—N11—C19—C20	−0.6 (6)
C6—W1—C1—N1	−145 (14)	Mn2—N11—C19—C20	175.2 (3)
C5—W1—C1—N1	−170 (100)	N11—C19—C20—C21	−0.2 (7)
Mn1^ii^—N2—C2—W1	9(10)	C19—C20—C21—C22	0.2 (7)
C4—W1—C2—N2	−77 (9)	C20—C21—C22—C23	0.6 (7)
C3—W1—C2—N2	141 (9)	C19—N11—C23—C22	1.4 (6)
C1—W1—C2—N2	179 (100)	Mn2—N11—C23—C22	−174.7 (3)
C7—W1—C2—N2	69 (9)	C19—N11—C23—C24	−176.1 (4)
C8—W1—C2—N2	−141 (9)	Mn2—N11—C23—C24	7.8 (4)
C6—W1—C2—N2	−11 (9)	C21—C22—C23—N11	−1.4 (6)
C5—W1—C2—N2	12 (9)	C21—C22—C23—C24	175.9 (4)
Mn2—N3—C3—W1	−10 (18)	C28—N12—C24—C25	−0.5 (6)
C2—W1—C3—N3	−4(14)	Mn2—N12—C24—C25	−178.3 (3)
C4—W1—C3—N3	−106 (14)	C28—N12—C24—C23	177.5 (3)
C1—W1—C3—N3	−162 (14)	Mn2—N12—C24—C23	−0.4 (4)
C7—W1—C3—N3	84 (14)	N11—C23—C24—N12	−4.9 (5)
C8—W1—C3—N3	−80 (14)	C22—C23—C24—N12	177.7 (4)
C6—W1—C3—N3	35 (14)	N11—C23—C24—C25	173.0 (4)
C5—W1—C3—N3	142 (14)	C22—C23—C24—C25	−4.4 (6)
Mn2^i^—N4—C4—W1	−21 (5)	N12—C24—C25—C26	0.5 (6)
C2—W1—C4—N4	118 (4)	C23—C24—C25—C26	−177.3 (4)
C3—W1—C4—N4	−152 (4)	C24—C25—C26—C27	0.1 (6)
C1—W1—C4—N4	−98 (4)	C25—C26—C27—C28	−0.7 (6)
C7—W1—C4—N4	11 (4)	C24—N12—C28—C27	−0.2 (6)
C8—W1—C4—N4	−178 (4)	Mn2—N12—C28—C27	177.6 (3)
C6—W1—C4—N4	54 (4)	C26—C27—C28—N12	0.7 (6)

Symmetry codes: (i) −*x*+3/2, *y*+1/2, −*z*+1/2; (ii) −*x*+3/2, *y*−1/2, −*z*+1/2.

### Hydrogen-bond geometry (Å, °)

**Table:**

*D*—H···*A*	*D*—H	H···*A*	*D*···*A*	*D*—H···*A*
O1—H1···O10^i^	0.84 (2)	2.02 (2)	2.822 (5)	162 (5)
O1—H2···O9^iii^	0.84 (2)	2.04 (3)	2.834 (4)	156 (5)
O2—H3···O6^iv^	0.83 (2)	1.87 (2)	2.703 (4)	173 (5)
O2—H4···N6^i^	0.82 (2)	2.25 (2)	3.052 (4)	165 (5)
O3—H6···O6^ii^	0.83 (2)	1.92 (2)	2.744 (4)	176 (5)
O3—H5···N5^ii^	0.82 (2)	2.48 (3)	3.238 (4)	153 (4)
O4—H8···O5^ii^	0.83 (2)	2.12 (2)	2.907 (5)	159 (4)
O4—H7···O9^v^	0.84 (2)	1.89 (2)	2.722 (4)	174 (4)
O5—H30···N6^vi^	0.84 (2)	2.13 (2)	2.946 (5)	164 (5)
O5—H29···O1^i^	0.84 (2)	2.32 (3)	3.111 (4)	157 (5)
O6—H32···O7	0.83 (2)	1.97 (2)	2.773 (4)	162 (5)
O6—H31···O5	0.83 (2)	1.97 (3)	2.753 (4)	157 (4)
O7—H34···N8^i^	0.82 (2)	2.02 (2)	2.810 (5)	159 (4)
O7—H33···N5	0.83 (2)	2.09 (2)	2.915 (5)	175 (4)
O8—H35···N7^vii^	0.83 (2)	2.03 (2)	2.840 (5)	165 (5)
O8—H36···O7	0.83 (2)	2.03 (2)	2.854 (4)	174 (5)
O9—H38···N6^vii^	0.84 (2)	2.66 (3)	3.372 (5)	143 (4)
O9—H37···O8	0.83 (2)	1.91 (2)	2.715 (5)	161 (5)

Symmetry codes: (i) −*x*+3/2, *y*+1/2, −*z*+1/2; (iii) *x*−1/2, −*y*+5/2, *z*+1/2; (iv) *x*−1, *y*, *z*; (ii) −*x*+3/2, *y*−1/2, −*z*+1/2; (v) −*x*+1, −*y*+2, −*z*; (vi) −*x*+5/2, *y*+1/2, −*z*+1/2; (vii) −*x*+2, −*y*+2, −*z*.
